# Falls in Functional Neurological Disorder: Prevalence, Risk Factors and Clinical Implications

**DOI:** 10.1111/ene.70665

**Published:** 2026-06-10

**Authors:** Zahra Mohammadi, Amirreza Keyvanfar, Rory Higgins, Jon Stone, Jan Coebergh, Mark J. Edwards, Glenn Nielsen

**Affiliations:** ^1^ Neuroscience and Cell Biology Research Institute City St George's, University of London London UK; ^2^ Infectious Diseases and Tropical Medicine Research Center Shahid Beheshti University of Medical Sciences Tehran Iran; ^3^ Toronto Rehabilitation Institute University Health Network Toronto Ontario Canada; ^4^ Centre for Clinical Brain Sciences Royal Infirmary of Edinburgh Edinburgh UK; ^5^ Department of Neurology Ashford St. Peter's Hospitals NHS Foundation Trust Chertsey UK; ^6^ Department of Neurology St. George's Hospital NHS Foundation Trust London UK; ^7^ Department of Basic and Clinical Neuroscience Institute of Psychiatry, Psychology and Neuroscience London UK; ^8^ Department of Neuropsychiatry Maudsley Hospital London UK

**Keywords:** accidental falls, FND, functional neurological symptom disorder, rehabilitation

## Abstract

**Background:**

Functional neurological disorder (FND) is a disabling condition often presenting with motor impairments, including gait disturbance and postural instability. Data on the prevalence and predictors of falls in people with FND are limited. This study aimed to investigate the prevalence of self‐reported falls among individuals with motor FND and identify factors associated with falling.

**Methods:**

Adults with motor FND were recruited from a tertiary neurology centre in London, UK, December 2021 to August 2024. Participants reported falls within the previous 6 months and completed assessments of sensory impairment, mobility, pain, fatigue, depression and anxiety. Group differences between recurrent fallers (≥ 2 falls) and those with one or no falls were examined, and logistic regression analyses identified predictors of recurrent falls.

**Results:**

One hundred participants (81% female, mean age 43.7 ± 14.2 years) were included. Falls were reported by 62%, and 56% reported recurrent falls. Among fallers, 64% reported injuries including bruising (39%), laceration (8%) and fracture (6%). Recurrent fallers had poorer plantar sensation and vibration detection, as well as worse scores for anxiety, depression, pain and functional mobility. A multivariable regression model indicated that absent plantar sensation (OR = 5.30, 95% CI [1.37, 20.44], *p* = 0.015), greater pain severity (OR = 1.25, 95% CI [1.05, 1.48], *p* = 0.011) and greater anxiety (OR = 1.09, 95% CI [1.004, 1.18], *p* = 0.040) were associated with increased odds of recurrent falls.

**Conclusion:**

Falls are common among individuals with motor FND. Absent plantar sensation, pain and anxiety were independently associated with recurrent falls, supporting a multidimensional approach to falls assessment and rehabilitation.

## 1 Introduction

Functional neurological disorder (FND) can present with a range of motor and non‐motor symptoms, such as limb weakness, gait disturbance, sensory impairment and seizures, which can substantially impact mobility and physical stability [1, 2]. These challenges can predispose individuals to falling. Furthermore, evidence suggests that psychological factors, including attention dysregulation, fear of falling and cognitive distraction, can exacerbate gait instability [3, 4]. Kinematic postural studies in FND have characterized this instability, demonstrating a common tendency for large amplitude trunk sway that paradoxically improves with redirection of attention [5, 6]. Clinically, these features are used as positive signs to support the diagnosis, for example a positive Functional Romberg test shows high amplitude sway with a tendency to fall toward support, usually without actually falling to the ground [7]. Despite these recognised vulnerabilities among people with FND, the prevalence and determinants of falls remain poorly understood.

Gait disturbance and falls are common complications of neurological conditions. In the context of Parkinson's disease, multiple sclerosis and stroke, the risk of falling is significantly higher than in the general population due to impairments in motor control, postural stability, sensory integration and cognitive function [8, 9]. Falls can lead to fractures and soft tissue injuries. Moreover, falls may have broader consequences, such as loss of self‐confidence, increased dependence, diminishing quality of life and impose a substantial burden on the health care system [10].

Understanding the prevalence and clinical manifestations of falls in people with motor FND is essential for recognising the scope of this problem and for identifying high‐risk individuals early in their treatment. This knowledge can guide the development of multidisciplinary rehabilitation that integrates patient education alongside physical and psychological interventions [11]. Current consensus recommendations for physiotherapy and occupational therapy for FND lack specific guidance regarding assessment and management of falls [12, 13]. Taken together, these gaps and challenges underscore the importance of accurate assessments that include fall history and associated risk factors during clinical evaluation. Therefore, this study aimed to determine the prevalence of self‐reported falls among individuals with motor FND and to identify factors associated with self‐reported falling in this population. By addressing this gap in the literature, we hope to encourage a more comprehensive and risk‐aware approach to managing falls in the context of FND.

## 2 Methods

### 2.1 Study Design, Setting and Participants

In this cross‐sectional study, individuals attending a specialised FND neurology clinic at St George's University Hospital in London, UK, from December 1, 2021, to August 8, 2024, were assessed for recruitment. The data are a subset of data from an observational study focused on sensory symptoms in people with motor FND [14].

Inclusion criteria were as follows: aged 18 years or older and a clinically definite diagnosis of FND according to the Gupta and Lang diagnostic criteria [15] with motor symptoms (e.g., functional weakness, gait disturbances and tremor). Exclusion criteria were: diagnosis of another condition that could be responsible for the presenting sensory dysfunction (e.g., peripheral neuropathy, stroke or multiple sclerosis), severe psychiatric disorders (e.g., severe anxiety, severe depression, current suicidal ideation), current compensation claims or lawsuits, an established learning disability that prevented the patient from completing the questionnaires independently, inability to comprehend English well enough to complete the study questionnaires and lack of capacity to give informed consent.

An information sheet and an explanation of the study were given to eligible participants. Those who gave consent to be contacted were called and asked to come to the hospital to take part in this study. Their eligibility was screened, and all completed written informed consent to participate.

### 2.2 Ethical Considerations

Ethical approval was obtained from the London Bloomsbury Research Ethics Committee (REC ref.: 21/LO/0724, November 12, 2021).

### 2.3 Outcome Measures and Data Collection

The primary outcome for this study was self‐reported fall history. Participants were asked whether they had experienced any falls in the previous 6 months and, if so, the number of falls experienced during that period. To distinguish between a fall and less severe balance disturbances, participants were also asked about near falls, defined as a loss of balance that would have resulted in a fall if a sufficient balance recovery response had not occurred [16].

Participants were categorised into three groups based on fall frequency over the previous 6 months: non‐fallers, single fallers and recurrent fallers, with recurrent falls defined as two or more falls. This categorization was informed by falls research recommendations to distinguish an isolated fall from recurrent fallers [16]. For inferential analyses, recurrent falls were selected as the primary outcome because repeated falls are more likely than isolated single falls to reflect an ongoing clinical problem. The comparison group for the primary analysis comprised participants with no falls or a single isolated fall. Participants were also asked about injuries sustained during falls.

In addition to fall frequency, we explored whether participants required assistance to stand following a fall as a descriptive indicator of both functional severity and the burden or consequences associated with falls.

We explored the associations between fall status and a range of demographic and clinical variables. Demographic variables included age, sex, ethnicity (which due to low numbers was collapsed into categories, white vs. non‐white), years of formal education and employment status (categorised as employed/student, not working due to ill health or other). Clinical variables included the duration of symptoms and pain severity, which was measured using a Visual Analogue Scale (VAS) ranging from 0 (no pain) to 10 (worst imaginable pain) [17].

Vibration detection was measured using a Rydel‐Seiffer tuning fork on the right and left first toe, at the bony prominence of the tarsometatarsal joint. This quantitative sensory test scores vibration intensity perception using a 64 Hz tuning fork on an ordinal scale of 0 to 8 (a score of 0 indicates absent vibration detection and 8 denotes intact vibration perception). Participants were instructed to indicate when they no longer perceived the decreasing vibration stimulus. Scores were averaged across three trials. Vibration detection threshold has been used in populations with FND to detect subtle sensory deficits that may contribute to postural instability and gait disturbances [18, 19].

Sensory function in the foot was assessed over the plantar surface of the first metatarsal head using Semmes‐Weinstein monofilaments (Touch‐Test 20 monofilament kit; North Coast Medical, USA). The testing procedure, described in detail elsewhere [14], followed a method of limits approach which categorised sensory function according to established clinical cut‐offs as follows: normal, diminished light touch, diminished protective sensation, loss of protective sensation, deep pressure sensation only and unable to feel/insensate [20, 21]. For regression analyses, responses were dichotomised into preserved versus absent monofilament detection to improve clinical interpretability, and also for statistical purposes, as responses clustered at the extremes with relatively few participants in intermediate sensory categories.

The Functional Mobility Scale assesses walking ability at three distances (5, 50 and 500 m), by rating the level of mobility aid assistance required. Each distance is scored from 1 to 6, leading to a total score range of 3 to 18. Higher scores indicate better mobility. Although it was originally designed for paediatric populations, its structure makes it adaptable for other clinical groups, including adults with FND [22].

Fatigue was assessed using the Vitality subscale of the Short Form‐36 (SF‐36) health survey. Scores range from 0 to 100, with lower scores indicating greater fatigue. The SF‐36 Vitality subscale is recognised as a reliable and valid measure of fatigue in neurological populations and other health conditions [23, 24].

Depression was assessed using the Patient Health Questionnaire‐9 (PHQ‐9), a widely used and validated self‐report measure for identifying and quantifying the severity of depression in both clinical and research settings [25]. Scores range from 0 to 27, with higher scores indicating greater severity of depression.

Anxiety was assessed using the Generalized Anxiety Disorder‐7 (GAD‐7) scale. Scores range from 0 to 21, with higher scores indicating more severe anxiety. The GAD‐7 has been validated in various clinical contexts and demonstrates acceptable psychometric properties for detecting anxiety symptoms in patients with chronic neurological disorders [26].

For the logistic regression analyses, variables were coded so that higher values consistently represented greater impairment (i.e., OR > 1 reflected increased odds of recurrent falls). Accordingly, for variables where higher scores originally reflected better function, the scales were reversed prior to analyses. The Functional Mobility Scale (range 3 to 18) was transformed using the formula (18 − original score + 3). Vibration detection and SF‐36 Fatigue were transformed using the formula (new score = max score − original score). Monofilament detection was coded as 1 = absent sensation and 0 = sensation present.

### 2.4 Statistical Analysis

Data were analysed using SPSS software version 31. Categorical variables were summarised with frequency and percentage. Continuous variables were summarised using mean and standard deviation (SD) or median and interquartile range (IQR), depending on their distributions. Normality assumptions were assessed by the Kolmogorov–Smirnov test for continuous variables before conducting inferential analysis.

Baseline demographic and clinical characteristics were compared between participants with and without recurrent falls (≥ 2 falls vs. 0–1 falls) using chi‐squared or Fisher's exact tests for categorical variables and independent‐samples *t*‐tests or Mann–Whitney *U* tests for continuous variables, as appropriate.

Multivariable logistic regression analysis was performed to determine predictors of recurrent falls. Initially, univariable logistic regression was used to estimate odds ratios (ORs) with 95% confidence intervals (CIs) for the association between each variable and recurrent fall status. Variables associated with recurrent falls in the univariable analysis (*p* < 0.10) were entered into a backward multivariable regression model (Wald method). For vibration and monofilament detection, only the side demonstrating the stronger univariable association with recurrent falls was entered into multivariable models to avoid multicollinearity and redundancy between bilateral measures assessing the same underlying sensory construct. At each step, variables with a *p*‐value above 0.05 were removed, and the model was refitted until only variables that were statistically significant at the 0.05 level remained. The final model included variables that were associated with recurrent falls and are considered predictors of recurrent falls.

Secondary analyses examined associations using any fall (rather than recurrent falls) as the outcome. Sensitivity analyses explored alternative multivariable model specifications, including substitution of depression scores for anxiety scores.

In secondary analyses, we identified a subgroup of frequent fallers, defined as ≥ 10 falls within 6 months, and explored whether this subgroup differed clinically from other participants. We also examined whether participants requiring assistance to stand after a fall differed from those able to stand independently. These analyses were considered exploratory and are reported descriptively.

A two‐sided *p*‐value of < 0.05 was considered statistically significant throughout the analysis. Box plots were generated using MATLAB R2024b (MathWorks, Natick, MA, USA).

## 3 Results

### 3.1 Baseline Characteristics of the Participants

A total of 102 individuals with a diagnosis of FND were included in the main dataset. Two participants were excluded due to missing data on the primary outcome (fall history), resulting in a final sample of 100. Among them, 38 (38%) reported no falls in the previous 6 months, 62 (62%) reported a history of at least one fall, 56 (56%) were recurrent fallers (2 or more falls), and 29 (29%) were frequent fallers (10 or more falls). Near falls (without falling) were reported by 13 (13%).

Among the 62 participants who reported falls, 40 (64.5%) sustained injuries as a result of their falls. The most common self‐reported injury was bruising (39%), followed by laceration (8%), fracture (6%), graze (4.9%), ankle sprain (3%), bumped head/minor head injury (3%), meniscus injury (3%) and dislocation (1.6%).

The mean age was 43.7 years (SD 14.2), with no significant difference between recurrent fallers and those with 0–1 falls (*p* = 0.673). The majority of participants were female (81%), with no significant difference in proportion between groups. Ethnicity and symptom duration did not differ between groups; there was a trend toward fewer years of education in the recurrent falls group (14.9 SD 3.1 vs. 16.0 SD 2.6 years, *p* = 0.051). Baseline characteristics are summarised in Table 1.

**TABLE 1:** Baseline characteristics of the participants by fall status.

Variables	Recurrent (≥ 2) falls (*n* = 56)	0–1 Falls (*n* = 44)	Total (*N* = 100)	*p*
Age (years), mean ± SD	44.3 ± 14.7	43.1 ± 13.5	43.7 ± 14.2	0.673^a^
Sex, *n* (%)				
Female	47 (83.9)	34 (77.3)	81 (81.0)	0.400^b^
Male	9 (16.1)	10 (22.7)	19 (19.0)	
Ethnicity, *n* (%)				
White	46 (83.6)	39 (88.6)	85 (85.9)	0.478^b^
Non‐white	9 (16.4)	5 (11.4)	14 (14.1)	
Years of education, mean ± SD	14.9 ± 3.1	16.0 ± 2.6	15.4 ± 2.9	0.051^a^
Employment, *n* (%)				
Employed/Student	21 (37.5)	23 (52.3)	44 (44.0)	0.089^b^
Not working due to ill health	12 (21.4)	12 (27.3)	24 (24.0)	
Other	23 (41.1)	9 (20.5)	32 (32.0)	
Symptom duration (years), median (IQR)	4.4 (1.8, 8.4)	4.1 (2.6, 7.4)	4.4 (2.2, 8.3)	0.789^c^
Dominant motor symptom presentation, *n* (%)				
Mixed movement disorder	22 (39.3)	11 (25.0)	33 (33.0)	0.304^d^
Weakness	11 (19.6)	13 (29.5)	24 (24.0)	
Gait disorder	14 (25.0)	8 (18.2)	22 (22.0)	
Tremor	5 (8.9)	5 (11.4)	10 (10.0)	
Dystonia	4 (7.1)	5 (11.4)	9 (9.0)	
Jerks	0 (0.0)	2 (4.5)	2 (2.0)	
PHQ‐9 (Depression) score, mean ± SD	14.4 ± 6.6	10.8 ± 5.4	12.8 ± 6.3	**0.005** ^a^
GAD‐7 (Anxiety) score, mean ± SD	10.8 ± 5.8	7.8 ± 5.6	9.5 ± 5.9	**0.013** ^a^
Pain score, median (IQR)	6.0 (5.0, 7.0)	4.0 (1.0, 7.0)	5.0 (4.0, 7.0)	**0.004** ^c^
Short Form 36 Fatigue, median (IQR)	18.8 (6.3, 37.8)	25.0 (12.5, 50.0)	25.0 (6.3, 42.2)	**0.040** ^c^
Functional Mobility Scale, median (IQR)	11.0 (6.0, 15.0)	15.0 (11.3, 18.0)	13.0 (8.3, 15.0)	**< 0.001** ^c^
Wheelchair used for most mobility, *n* (%)	5 (8.9)	4 (9.1)	9 (9.0)	1.000^e^
Vibration detection, right, median (IQR)	7.3 (4.0, 8.0)	8.0 (6.0, 8.0)	8.0 (5.3, 8.0)	**0.047** ^c^
Vibration detection, left, median (IQR)	6.0 (0.0, 8.0)	8.0 (6.0, 8.0)	7.8 (4.1, 8.0)	**0.012** ^c^
Unable to detect monofilament (right), *n* (%)	11 (19.6)	3 (6.8)	14 (14.0)	0.067^b^
Unable to detect monofilament (left), *n* (%)	19 (33.9)	3 (6.8)	22 (22.0)	**0.001** ^b^

*Note:* PHQ‐9 score range 0–27; GAD‐7 score range 0–21; Pain score visual analogue scale range 0–10; Short Form 36 Fatigue/Vitality score range 0–100 (lower scores indicate worse fatigue); Functional Mobility Scale range 3–18 (lower scores indicate worse mobility); Vibration detection score range 0–8 (lower scores indicate worse vibration sense). Wheelchair used for most mobility was defined as wheelchair use across all Functional Mobility Scale distances: 5, 50 and 500 m. The bold values indicate the statistically significant *p* values.

^a^ Independent‐samples *t*‐test.

^b^ Chi‐squared test.

^c^ Mann–Whitney *U* test.

^d^ Fisher–Freeman–Halton exact test with Monte Carlo estimation.

^e^ Fisher's exact test.

### 3.2 Clinical Assessments

**FIGURE 1:**
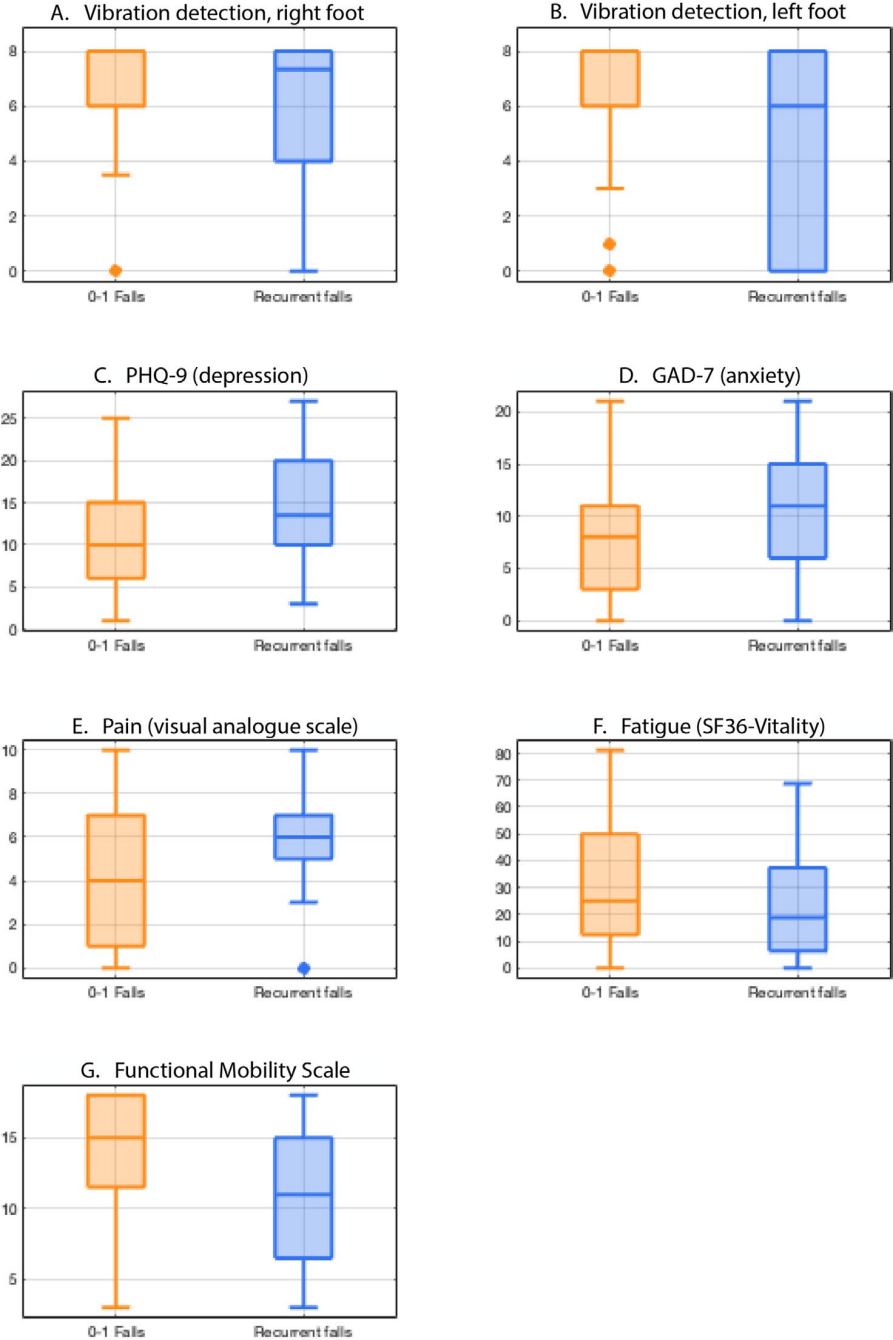
Box‐plot comparison of the clinical, sensory, psychological and functional assessments between participants with 0–1 falls (*n* = 44) and recurrent (≥ 2) falls (*n* = 56). Vibration detection (score range 0–8, lower scores indicate worse vibration sense); PHQ‐9 = Patient Health Questionnaire‐9 (score range 0–27); GAD‐7 = Generalised Anxiety Disorder‐7 (score range 0–21); Pain visual analogue scale (score range 0–10); SF‐36 = Short Form 36 Health Survey Fatigue/Vitality (score range 0–100, lower scores indicate worse fatigue); Functional Mobility Scale (score range 3–18, lower scores indicate worse mobility).

Figure 1 illustrates differences in clinical measures between recurrent fallers and the 0–1 falls group. Recurrent fallers exhibited worse vibration detection and were more likely to have absent monofilament detection. They also reported greater pain, fatigue, depression, anxiety and lower functional mobility scores. Detailed values are presented in Table 1.

### 3.3 Logistic Regression Models

Table 2 presents the results of logistic regression analyses examining predictors of recurrent falls. In univariable analysis, worse scores for sensory function (vibration and monofilament detection), mobility, pain, depression, anxiety and fatigue were each associated with increased odds of recurrent falls.

**TABLE 2:** Logistic regression analysis to identify predictors of recurrent falls in patients with FND.

Variable	Univariable model		Multivariable model	
	OR (95% CI)	*p*	OR (95% CI)	*p*
Age (years)	1.00 (0.98, 1.04)	0.670	—	
Female sex	1.54 (0.56, 4.19)	0.402	—	
Years of education	0.87 (0.76, 1.00)	0.055	—	
Symptom duration	1.01 (0.96, 1.06)	0.662	—	
Vibration detection right foot	1.18 (1.01, 1.37)	**0.038**	—	
Vibration detection left foot	1.20 (1.03, 1.38)	**0.016**	—	
Monofilament detection right foot	3.34 (0.87, 12.82)	**0.079**	—	
Monofilament detection left foot	7.02 (1.92, 25.65)	**0.003**	5.30 (1.37, 20.44)	**0.015**
Functional Mobility Scale	1.15 (1.05, 1.27)	**0.002**	—	
PHQ‐9 (Depression)	1.10 (1.03, 1.18)	**0.007**	—	
GAD‐7 (Anxiety)	1.09 (1.02, 1.18)	**0.016**	1.09 (1.004, 1.18)	**0.040**
Short Form 36 Fatigue score	1.02 (1.00, 1.04)	**0.024**	—	
Pain Visual Analogue Scale	1.29 (1.10, 1.52)	**0.002**	1.25 (1.05, 1.48)	**0.011**

*Note:* Variables were coded such that higher values represent greater impairment. For variables where higher scores originally reflected better function (e.g., fatigue, mobility and vibration detection), scales were reversed prior to analysis. Multivariable model derived using backward stepwise logistic regression including variables with *p* < 0.10 in univariable analysis. Only left‐sided measures were included in multivariable analysis due to collinearity. The bold values indicate the statistically significant *p* values.

In the multivariable logistic regression model, impaired monofilament detection (OR = 5.30, 95% CI [1.37, 20.44], *p* = 0.015), higher pain scores (OR = 1.25, 95% CI [1.05, 1.48], *p* = 0.011) and higher anxiety scores (OR = 1.09, 95% CI [1.004, 1.18], *p* = 0.040) remained significantly associated with increased odds of recurrent falls after adjusting for other variables. Absent sensation on monofilament detection was associated with 5 times higher odds of recurrent falls; each one‐point increase in pain VAS score was associated with a 25% increase in the odds of recurrent falls, and each one‐point increase in anxiety score was associated with a 9% increase in the odds of recurrent falls.

### 3.4 Sensitivity and Secondary Analyses

In a sensitivity analysis, a reduced model including only monofilament detection, pain and anxiety yielded similar effect sizes and statistical significance to the primary model (Table S1).

In a secondary analysis using multivariable logistic regression comparing all fallers versus non‐fallers, associations between sensory impairment and falls were attenuated and no longer statistically significant, although trends in the same direction were observed. Pain severity remained independently associated with falls, while worse Functional Mobility Scale scores also emerged as a significant predictor (Table S2).

### 3.5 Exploratory Subgroup Analysis

In exploratory analyses, participants with frequent falls (≥ 10 falls) demonstrated worse left‐sided vibration detection (*p* = 0.005) and were more likely to have absent monofilament detection in the left foot (*p* = 0.014) compared to those with fewer falls. There was also a trend toward poorer mobility and longer symptom duration in frequent fallers, although these did not reach statistical significance. No differences were observed in pain, anxiety, depression or fatigue scores (Table S3).

Among participants who reported at least one fall, those who required assistance to stand after falling were more likely to have absent monofilament sensation in both feet (*p* = 0.007 right; *p* = 0.001 left) compared to those able to stand independently. They also reported higher pain scores (*p* = 0.021), greater depressive symptoms (*p* = 0.035) and increased fatigue (*p* = 0.010) (Table S4). These subgroup analyses were exploratory and unadjusted for potential confounders and should be interpreted with caution.

## 4 Discussion

Falls are a well‐recognised source of morbidity and mortality in neurological conditions, but they remain under‐researched in motor FND and are often assumed to be unimportant. However, our results challenge this assumption, highlighting that falls in individuals with FND are frequent, concerning and linked to potentially modifiable sensory and psychological mechanisms.

In our cohort, 62% of individuals with motor FND reported a fall in the previous 6 months and 56% experienced recurrent falls, defined as two or more falls within 6 months. Factors independently associated with recurrent falls were absent plantar monofilament detection, pain severity and anxiety. Overall, the findings emphasise the complex and multifactorial nature of fall risk in people with FND. Falls appear to result from an interaction between sensorimotor dysfunction, symptom perception, mobility limitations and psychological factors.

The strongest predictor of recurrent falling was absent plantar monofilament detection. Participants classified as having absent sensation were unable to detect the highest force monofilament in the testing kit (300 g), indicating absence of plantar light touch and deep pressure sensation [27]. This represents a novel finding supporting the hypothesis that sensory impairment contributes to fall risk in motor FND. Plantar sensory feedback plays a critical role in postural control, gait adaptation and detection of shifts in body position, and impaired plantar sensation is associated with falls in neurological and non‐neurological conditions [28, 29, 30]. Our findings challenge assumptions that sensory symptoms in FND are purely attention dependent and context specific and suggest that when sensory loss is clinically present it may nevertheless have functionally important consequences for mobility and balance. One possible explanation is that altered sensory processing or integration in FND reduces the reliability of afferent feedback required for postural control, thereby increasing instability and fall risk. Reduced sensory feedback may also alter behavioural responses to balance perturbations, including protective stepping or corrective postural reactions, potentially contributing to maladaptive balance strategies and increased falls susceptibility. These findings support the inclusion of sensory assessment in falls evaluation and rehabilitation planning for motor FND.

Pain severity was the second strongest predictor of recurrent falls. Each one‐point increase in pain VAS score was associated with a 25% increase in the odds of recurrent falls. Pain can interfere with balance and gait through several mechanisms. It diverts the attentional resources required for postural control, promotes adaptive gait patterns to avoid discomfort (e.g., limping or asymmetrical step timing), and induces avoidance behaviours that lead to deconditioning. This is highly relevant to FND, as previous studies have shown that many individuals with FND experience widespread pain that interferes with daily functioning [31]. Pain‐related gait adaptations are well documented. For instance, individuals with musculoskeletal pain often shorten their stride, reduce walking speed and shift their weight to avoid pressure on a painful limb, all of which can compromise dynamic stability [32]. Similar cautious, asymmetric or unsteady gait patterns are observed in FND, even when normal power is present during clinical examination. The clinical implications are clear, pain should be actively assessed and addressed as a potential contributor to disability and falls. Interventions such as graded activity, pain neuroscience education and cognitive‐behavioural strategies are valuable additions to rehabilitation programs for reducing pain‐related motor disruption [33].

The final predictor of recurrent falls in the multivariable model was higher anxiety. Each one‐point increase in GAD‐7 score was associated with a 9% increase in the odds of recurrent falls. One potential explanation for this association is the interaction between anxiety, fear of falling and postural control. While fear can be protective, the Perceived Control Model of Falling proposes that the protective nature of fear becomes maladaptive when individuals perceive low overall control in situations that threaten their balance [34]. According to this model, low perceived control combined with a heightened emotional response to balance perturbations can trigger panic and rigid, maladaptive postural responses. Although the model was developed with older adults in mind, postural instability and falls in FND may represent a clinical manifestation of this model. The shoulder‐tap test has been proposed as a clinical sign of functional gait disorder that evokes abnormal anticipatory postural reactions to perceived balance threat [35]. With this test, patients with functional gait disorders demonstrated excessive stepping, exaggerated sway, startle‐like reactions and falls following a light shoulder tap that would not normally destabilise posture. These responses were interpreted as manifestations of postural hypervigilance and heightened anticipatory behaviour, rather than impaired postural reflexes. Anxiety may therefore contribute to falls in FND not only through emotional distress, but also through altered attentional focus, heightened threat monitoring and disruption of normally automatic balance control mechanisms.

Dissociation may represent another mechanism contributing to falls in FND [36, 37]. Hoeritzauer et al. [36] revisited the concept of cryptogenic drop attacks, which were originally defined as a fall without an obvious cause or warning, and not associated with a clear loss of consciousness or vertigo. The authors conducted a retrospective review and found that the majority of cases had comorbid FND or a functional somatic syndrome. It was proposed that cryptogenic drop attacks may reflect brief dissociative episodes that may be conceptualised as brief functional seizures overlapping with sudden functional leg weakness. It was suggested that, in some individuals, they may represent a conditioned response to a previous fall. Subjects were typically able to get up quickly unless they were injured but often reported being worried about future attacks. In our clinical practice we have in some rare specific patients considered if repeated falls might be conceptualized as a form of indirect or non‐conscious self‐harm, occurring in the context of dissociation, emotional distress and reduced interoceptive awareness, however we do not have clear evidence to support this hypothesis.

Falls in FND are often described as controlled events, typically presenting as postural instability with near misses or slow descents [1]. Posturographic analysis by Wolfsegger et al. [5] found that while participants with FND exhibited significantly higher trunk sway than healthy controls and those with multiple sclerosis, they demonstrated paradoxical improvement in stability when distracted. Stins et al. [6] proposed that this instability arises from excessive inward focus of attention and the maladaptive use of voluntary motor control strategies. One hypothesis arising from this work is that individuals with FND tend to fall frequently because they attend excessively to balance and posture in a way that impairs it, but once the fall commences, automatic postural and protective reflexes tend to lessen injury.

However, our study and the findings from Hoeritzauer et al. [36] challenge an assumption that falls are necessarily controlled and benign manifestations of FND. In the current study, 64% of fallers (representing 40% of the total motor FND cohort) reported experiencing fall‐related injuries. Similarly, Hoeritzauer et al. noted cryptogenic drop attacks were associated with soft tissue injuries in 29% of cases (24/83) and fractures in 9% (8/83). More than half of those with soft tissue injuries experienced recurrent facial injuries, indicating an absence of a face protective upper limb response. While most injuries are relatively minor, the risks present a challenge in rehabilitation, where the potential for injury must be weighed against the cost of overly risk‐averse mobility restrictions. A supported risk‐evaluation and positive risk‐taking approach is required, which can be delivered in multidisciplinary rehabilitation, involving physiotherapy, occupational therapy and psychology [12, 13, 38]. Recommendations for considering falls in rehabilitation are provided in Box 1.

BOX 1 **Recommendations for assessment and treatment of falls in functional neurological disorder.**

**Table:** 

Recommendations for managing falls in individuals with FND and motor symptoms
These clinical recommendations were informed by World Guidelines for Falls Prevention and Management for Older Adults [39], as well as consensus recommendations for physiotherapy [12] and occupational therapy [13] practice for FND.
**1. Assessment**
Clinicians should routinely ask individuals with FND (including motor symptoms and seizures) about falls. Key areas to assess include:
Frequency and type of falls: differentiate between near‐falls, controlled descents and sudden collapses without warning. Sudden uncontrolled drops carry higher injury risk and warrant consideration for alternative pathology (e.g. cardiac and vestibular causes).
Mechanism: trips, loss of balance, leg weakness, dissociation, sudden giving‐way or falls without a clear precipitant.
Warning signs or prodrome: dizziness, dissociation, symptoms of panic, visual blurring, etc. These may inform management strategies.
After a fall: what assistance is required to stand and continue with the day?
**2. Screening for co‐existing health conditions**
FND commonly coexists with other conditions which may contribute to risk of falling
Commonly overlooked and/or under‐treated conditions that may contribute to falls risk include visual impairment, migraine, cardiovascular disease, vestibular disorders, peripheral neuropathy and generalised anxiety disorder.
Consider the impact of medication use and polypharmacy on falls risk.
Specific FND symptoms and commonly co‐occurring syndromes may contribute to falls and are amendable to specific treatments. These include dizziness (persistent postural perceptual dizziness), functional cognitive disorder and postural orthostatic tachycardia syndrome.
Consider risk of fractures associated with osteoporosis and assess/treat as appropriate, especially in individuals who have had long periods without upright weightbearing and those with a history of nutritional compromise.
Consider nutritional status, including alcohol intake and adequate hydration, especially in individuals with bladder impairment who may have limited fluid intake.
**3. Addressing anxiety, fear of falling and panic**
Fear of falling, panic symptoms and heightened autonomic arousal increase the risk of falling and contribute to activity avoidance and deconditioning.
Psychological assessment and treatment of anxiety, depression and other mental health problems as appropriate. This may include self‐harming behaviour. Cognitive Behavioural Therapy may be particularly helpful for fear of falling and avoidance behaviour.
Psychologically informed rehabilitation: All clinicians can incorporate strategies such as:
Challenging unhelpful beliefs about falling
Goal setting and graded exposure to walking, balance practice and daily activities
Techniques to regulate anxiety, panic and dissociation during rehabilitation (e.g. controlled breathing and sensory grounding strategies)
**4. Encourage positive risk taking as part of the rehabilitation approach**
Supporting safe, positive risk taking is central to rehabilitation and gait and balance rehabilitation.
Encourage collaborative decision making between the patient and multidisciplinary team to balance the risk of injury with the risk of excessive safety behaviours restricting mobility and exacerbating falls risk.
Identify patterns of avoidance which may be contributing to falls that can be addressed in rehabilitation with support and graded exposure.
Involve family members and carers to support building confidence and improved mobility.
**5. Psychologically informed physical rehabilitation**
Rehabilitation should aim to restore movement control and independence while integrating principles that address panic, dissociation and symptoms or mechanisms associated with disability. This can include:
Gait retraining and balance rehabilitation: restoring fluent, automatic movement rather than over‐controlled or effortful gait patterns. A body weight support harness may be useful to commence rehabilitation for individuals with uncontrolled sudden falls.
Building insight: helping patients understand links between panic, altered balance responses, postural instability and falls.
Regulation strategies during movement: For example, practicing ‘pause, breathe, reset’ strategies when symptoms escalate.
Floor transfers: assessing and teaching the ability to get on and off the floor independently and daily practice of this action can reduce fear of being unable to rise after a fall.
Functional practice: incorporating balance and stability strategies into real‐life activities (e.g. meal preparation, showering, reaching and household tasks).
General physical activity: encouraging regular, enjoyable, non‐specific exercise and incidental movement to counteract deconditioning.
**6. Use of mobility aids**
Mobility aids can increase participation and confidence but may reduce natural balance responses and reinforce fear or dependence if overused, especially during acute phase of rehabilitation.
Provide balanced information about benefits and potential downsides of mobility aids and avoid excessive or unnecessary use.
Encourage flexible, graded weaning. For example, using aids on difficult days or in challenging environments, but gradually increasing unassisted walking as able.
Support patients in identifying when unaided walking is safe and when an aid remains helpful.
If prescribing aids, help the individual plan regular opportunities to practice safe mobility without them, and implement strategies to minimise over‐reliance and secondary joint pain or injury.
**7. Environmental access and participation**
Identify and mitigate environmental hazards in the home (e.g. loose rugs, poor lighting, clutter).
Consider adaptations to reduce activity avoidance and support independence (e.g. adaptations to assist independent washing, dressing and meal preparation).
Support ongoing employment and education, consider vocational rehabilitation specialists and advocate for reasonable adaptations.
Encourage engagement in daily activities that optimise quality of life while maintaining mobility and independence.

This study has several strengths. It focused on a well‐defined cohort of patients with motor FND which enhances the generalisability of the findings to real‐world practice. Additionally, the assessment of a wide range of factors, including sensory function, anxiety, mood, fatigue, pain and mobility, provided a comprehensive, holistic view of potential contributors to falls. The study has several limitations. The cross‐sectional design precludes the determination of causal relationship or temporal precedence between falls and the associated factors. For instance, it remains unclear whether reduced mobility leads to falls or whether falls subsequently result in mobility avoidance. The absence of a control group, either healthy controls or another clinical population, limits conclusions about the specificity of these findings to FND, including whether falls are more frequent or have distinct clinical correlates compared with other groups. Unmeasured confounding factors, such as cognitive impairment and polypharmacy, may have influenced falls risk. Nine participants used a wheelchair for most mobility, which may have reduced exposure to walking‐related falls and influenced fall reporting. However, wheelchair use was balanced between the groups, and five of these nine participants were recurrent fallers, indicating that wheelchair use did not preclude falling. Furthermore, fall history and resulting injuries were based on self‐report, which is susceptible to error and recall bias, including memory inaccuracy, or potential over‐reporting (e.g., interpreting a near fall as a fall) or under‐reporting. Future research should consider prospective designs with standardised assessments of medication use, cognition, fear of falling and other factors previously associated with falling in other populations.

## 5 Conclusion

In summary, falls are common among individuals with motor FND. Impaired plantar sensation, greater pain severity and anxiety emerged as significant predictors of recurrent falls in a multivariable model. Fall‐related injuries were common, although severe orthopaedic injuries are relatively uncommon. The consequences of falling in motor FND extend beyond injury‐related morbidity and may have a greater impact by increasing fear of falling and promoting maladaptive responses, thereby perpetuating instability and disability. These findings emphasize the importance of comprehensive, multidisciplinary assessment across psychological, physical and sensory domains, alongside assessment of pain. Addressing falls in FND should therefore be considered a clinical priority and a target for rehabilitation.

## Author Contributions

**Mark J. Edwards:** conceptualization, funding acquisition, writing – review and editing, supervision. **Glenn Nielsen:** conceptualization, investigation, funding acquisition, writing – review and editing, formal analysis, supervision. **Rory Higgins:** conceptualization, investigation, writing – review and editing, project administration, data curation. **Zahra Mohammadi:** writing – original draft, writing – review and editing, formal analysis. **Jan Coebergh:** conceptualization, writing – review and editing, investigation. **Jon Stone:** conceptualization, funding acquisition, writing – review and editing, supervision. **Amirreza Keyvanfar:** writing – review and editing, formal analysis.

## Funding

Glenn Nielsen is funded by Health Education England (HEE)/NIHR ICA Programme Clinical Lectureship NIHR301263 for this research project. The views expressed are those of the authors and not necessarily those of the NIHR, NHS or the Department of Health and Social. The research was also supported by the NIHR South London Clinical Research Network.

## Conflicts of Interest

J.S. reports honoraria from UptoDate, personal fees from Expert Witness Work and grants from National Research Scotland. He runs a free self‐help website, www.neurosymptoms.org, for patients with Functional Neurological Disorder. He is secretary of the FND Society and on the medical advisory boards of FND Action and Fowlers Syndrome UK. J.C. does medical expert reporting in personal injury and clinical negligence cases, including in cases of FND. He has received speaker fees from Merck, Orphalan, Novartis and Bial in the last 2 years. M.J.E. does medical expert reporting in personal injury and clinical negligence cases, including in cases of functional neurological disorder (FND). M.J.E. has shares in Brain & Mind, which provides neuropsychiatric and neurological rehabilitation in the independent medical sector, including in people with functional neurological disorder. M.J.E. has received financial support for lectures from the International Parkinson's and Movement Disorders Society and the FND Society (FNDS). M.J.E. receives royalties from Oxford University Press for his book The Oxford Specialist Handbook of Parkinson's Disease and Other Movement Disorder. M.J.E. has received honoraria for medical advice to Teva Pharmaceuticals. M.J.E. is deputy editor of the European Journal of Neurology. M.J.E. is on the medical advisory boards of the charities FND Hope and Dystonia UK. G.N. is a founding member and on the board of directors of the Functional Neurological Disorders Society. He is on the medical advisory boards of the charities FND Hope UK and FND Action. The other authors declare no conflicts of interest.

## Supporting information


**Table S1:** Sensitivity analysis for predictors of recurrent falls with reduced model.
**Table S2:** Secondary analysis comparing all fallers (*n* = 62) versus non‐fallers (*n* = 38).
**Table S3:** Exploratory subgroup analysis comparing frequent fallers (≥ 10 falls over 6 months) with other participants.
**Table S4:** Exploratory subgroup analysis comparing fallers requiring assistance to stand after a fall versus those able to stand independently.

## Data Availability

Deidentified participant data can be made available by request to the corresponding author. Requests will be considered after planned analyses and reporting have been completed by the investigators. Access will require submission of a protocol that is approved by a review committee.
