# Supplementary material for: Falls in Functional Neurological Disorder: Prevalence, Risk Factors and Clinical Implications
Source: Eur J Neurol. 2026 Jun 10;33(6):e70665. doi: 10.1111/ene.70665 (PMC13250761; doi:10.1111/ene.70665)
Supplement: Supplementary file 1 — Table S1: Sensitivity analysis for predictors of recurrent falls with reduced model. Table S2: Secondary analysis comparing all fallers (n = 62) versus non‐fallers (n = 38). Table S3: Exploratory subgroup analysis comparing frequent fallers (≥ 10 falls over 6 months) with other participants. Table S4: Exploratory subgroup analysis comparing fallers requiring assistance to stand after a fall versus those able to stand independently. [file ENE-33-e70665-s001.docx]

# APPENDIX

**Table S1.** Sensitivity analysis for predictors of recurrent falls with reduced model

| **Model** | **Variable** | **OR (95% CI)** | **p-value** |
| --- | --- | --- | --- |
| Reduced model | Monofilament detection (absent vs present) | 5.30 (1.37–20.44) | 0.015 |
|  | Pain (Visual Analogue Scale) | 1.25 (1.05–1.48) | 0.011 |
|  | GAD-7 (Anxiety) | 1.09 (1.00–1.18) | 0.040 |

**Table S2.** Secondary analysis comparing all fallers (n=62) versus non-fallers (n=38)

| **Variable** | **Univariable model** | | **Multivariable model** | |
| --- | --- | --- | --- | --- |
|  | **OR (95% CI)** | **p-value** | **OR (95% CI)** | **p-value** |
| Age (years) | 1.00 (0.98, 1.03) | 0.767 | - |  |
|  |  |  |  |  |
| Female sex | 2.10 (0.77-5.78) | 0.149 | - |  |
|  |  |  |  |  |
| Years of education | 0.94 (0.81, 1.07) | 0.340 | - |  |
|  |  |  |  |  |
| Symptom duration | 1.00 (0.95, 1.05) | 0.969 | - |  |
|  |  |  |  |  |
| Vibration detection right foot | 1.21 (1.02, 1.43) | **0.029** | - |  |
| Vibration detection left foot | 1.18 (1.01, 1.37) | **0.034** | - |  |
|  |  |  |  |  |
| Monofilament detection right foot | 4.32 (0.91, 20.50) | 0.065 | - |  |
| Monofilament detection left foot | 5.16 (1.41, 18.86) | **0.013** | - |  |
|  |  |  |  |  |
| Functional Mobility Scale | 1.18 (1.06, 1.30) | **0.002** | 1.14 (1.03, 1.27) | **0.013** |
|  |  |  |  |  |
| PHQ-9 (Depression) | 1.09 (1.01, 1.17) | **0.021** | - |  |
|  |  |  |  |  |
| GAD-7 (Anxiety) | 1.07 (0.99, 1.15) | 0.072 | - |  |
|  |  |  |  |  |
| Short Form 36 Fatigue score | 1.03 (1.01, 1.05) | **0.008** | 1.02 (1.00, 1.05) | 0.058 |
|  |  |  |  |  |
| Pain Visual Analogue Scale | 1.36 (1.14, 1.61) | **<0.001** | 1.24 (1.04, 1.49) | **0.020** |

**Table S3.** Exploratory subgroup analysis comparing frequent fallers (≥10 falls over 6 months) with other participants

| **Variable** | **Frequent fallers (n=29)** | **Others (n=71)** | **p-value** |
| --- | --- | --- | --- |
| Vibration detection, right, median (IQR) | 7.0 (4.0, 8.0) | 8.0 (6.0, 8.0) | 0.159 |
| Vibration detection, left, median (IQR) | 6.0 (0.0, 8.0) | 8.0 (5.9, 8.0) | 0.005 |
| Pain, median (IQR) | 6.0 (4.5, 7.0) | 5.0 (3.0, 7.0) | 0.128 |
| SF-36 Fatigue, median (IQR) | 18.8 (9.4, 37.5) | 25.0 (6.3, 45.3) | 0.848 |
| Functional Mobility Scale, median (IQR) | 11.0 (7.0, 15.0) | 14.5 (9.0, 16.3) | 0.053 |
| Symptom duration, median (IQR) | 5.5 (3.1, 10.8) | 3.9 (1.6, 7.3) | 0.098 |
| Unable to detect monofilament, right, n (%) | 6 (20.7%) | 8 (11.3%) | 0.222 |
| Unable to detect monofilament, left, n (%) | 11 (37.9%) | 11 (15.5%) | 0.014 |

Continuous variables were compared using Mann–Whitney U tests and categorical variables using chi-square or Fisher’s exact tests, as appropriate.

**Table S4.** Exploratory subgroup analysis comparing fallers requiring assistance to stand after a fall versus those able to stand independently

| **Variable** | **Independent stand (n=31)** | **Required assistance to stand (n=30)** | **p-value** |
| --- | --- | --- | --- |
| Vibration detection, right, median (IQR) | 7.0 (4.3, 8.0) | 7.7 (0.0, 8.0) | 0.813 |
| Vibration detection, left, median (IQR) | 7.0 (5.3, 8.0) | 5.8 (0.0, 8.0) | 0.207 |
| Pain, median (IQR) | 6.0 (4.0, 7.0) | 7.0 (5.0, 8.0) | 0.021 |
| SF-36 Fatigue, median (IQR) | 25.0 (12.5, 37.5) | 12.5 (6.3, 25.0) | 0.010 |
| Functional Mobility Scale, median (IQR) | 12.0 (8.0, 15.0) | 9.0 (4.8, 15.0) | 0.085 |
| Symptom duration, median (IQR) | 4.4 (2.8, 8.3) | 3.1 (1.1, 8.0) | 0.120 |
| Unable to detect monofilament, right, n (%) | 2 (6.3%) | 10 (33.3%) | 0.007 |
| Unable to detect monofilament, left, n (%) | 4 (12.5%) | 15 (50.0%) | 0.001 |

Continuous variables were compared using Mann–Whitney U tests and categorical variables using chi-square or Fisher’s exact tests, as appropriate.
